# Improving Intra-Fractional Target Position Accuracy Using a 3D Surface Surrogate for Left Breast Irradiation Using the Respiratory-Gated Deep-Inspiration Breath-Hold Technique

**DOI:** 10.1371/journal.pone.0097933

**Published:** 2014-05-22

**Authors:** Yi Rong, Steve Walston, Meng Xu Welliver, Arnab Chakravarti, Allison M. Quick

**Affiliations:** Department of Radiation Oncology, The Ohio State University Wexner Medical Center, The James Cancer Hospital, Columbus, Ohio, United States of America; University of California Davis, United States of America

## Abstract

**Purpose:**

To evaluate the use of 3D optical surface imaging as a surrogate for respiratory gated deep-inspiration breath-hold (DIBH) for left breast irradiation.

**Material and Methods:**

Patients with left-sided breast cancer treated with lumpectomy or mastectomy were selected as candidates for DIBH treatment for their external beam radiation therapy. Treatment plans were created on both free breathing (FB) and DIBH computed tomography (CT) simulation scans to determine dosimetric benefits from DIBH. The Real-time Position Management (RPM) system was used to acquire patient's breathing trace during DIBH CT acquisition and treatment delivery. The reference 3D surface models from FB and DIBH CT scans were generated and transferred to the “AlignRT” system for patient positioning and real-time treatment monitoring. MV Cine images were acquired during treatment for each beam as quality assurance for intra-fractional position verification. The chest wall excursions measured on these images were used to define the actual target position during treatment, and to investigate the accuracy and reproducibility of RPM and AlignRT.

**Results:**

Reduction in heart dose can be achieved using DIBH for left breast/chest wall radiation. RPM was shown to have inferior correlation with the actual target position, as determined by the MV Cine imaging. Therefore, RPM alone may not be an adequate surrogate in defining the breath-hold level. Alternatively, the AlignRT surface imaging demonstrated a superior correlation with the actual target positioning during DIBH. Both the vertical and magnitude real-time deltas (RTDs) reported by AlignRT can be used as the gating parameter, with a recommended threshold of ±3 mm and 5 mm, respectively.

**Conclusion:**

The RPM system alone may not be sufficient for the required level of accuracy in left-sided breast/CW DIBH treatments. The 3D surface imaging can be used to ensure patient setup and monitor inter- and intra- fractional motions. Furthermore, the target position accuracy during DIBH treatment can be improved by AlignRT as a superior surrogate, in addition to the RPM system.

## Introduction

Adjuvant radiotherapy (RT) after lumpectomy or mastectomy significantly reduces the risk of local cancer recurrence and cancer-related mortality [1], [2]. However, radiation exposure to the heart and normal lung may result in late radiation-induced cardiac and pulmonary complications, including heart disease and lung cancer [3]–[5]. Studies have shown that a linear relationship exists between mean heart dose and increased rates of chest pain, coronary artery disease and myocardial infarction for women treated with radiation for left-sided breast cancer [6]–[8]. Cardiac toxicity has been implicated as one of the major factors responsible for reduced overall survival, especially when radiation is given to the ipsilateral internal mammary and periclavicular lymph nodes [9], [10].

Deep inspiration breath hold (DIBH) is a technique that can reduce the volume of heart receiving radiation by increasing the distance between the heart and the breast or chest-wall (CW) [11]–[16]. DIBH can be done using two different methods, voluntary DIBH or moderate DIBH. For voluntary DIBH, patients are instructed on deep inspiration and the respiratory motion is tracked by an external surrogate, i.e. Real-time Position Management (RPM) system (Varian Medical Systems, Palo Alto, CA) [15], [17], [18]. The RPM system monitors the vertical displacement of the sternum or abdomen and provides a relative value with respect to the patient's breathing baseline [19]. Moderate DIBH uses an active breathing control (ABC) device (Elekta Oncology Systems, Stockholm, Sweden) to actively control the lung expansion at a user-defined volume, usually 75% of the maximum inspiration capacity, during the beam-on time [13], [20]. The ABC device moderates the breathing cycle by controlling the lung volume. However, neither of these techniques can monitor or gate treatment based on the breast or CW position, which may result in less reliable dose delivery to the target. Alternatively, the combined use of respiratory-gated DIBH and an active surface monitoring system may improve treatment accuracy and reproducibility by actively monitoring the target motion.

Optical tracking systems, such as the AlignRT system (Vision RT Ltd, London, UK), have recently been introduced in radiotherapy [21]–[23]. The AlignRT system employs stereo vision technology by viewing an object through three pods, each containing two cameras, and reconstructs the 3D surface information of the object using a computer vision algorithm. This system can be used for patient positioning prior to treatment, real-time target monitoring, and respiratory tracking. Six degrees of alignment between the reference surface model (RSM) and the reconstructed surface imaging are displayed in real-time for the contoured regions of interests (ROIs). Several investigators have studied the feasibility and accuracy of the AlignRT system for brain stereotactic radiotherapy (SRS), as well as the treatment of breast and lung cancers [24]–[31]. Among the recent publications on the use of AlignRT for the left-breast DIBH treatment, three focused on patient setup accuracy prior to treatment using AlignRT, as compared to multiple on-board imaging modalities, including weekly port films [29] and cone beam CTs (CBCTs) [27], [28]. Two additional studies investigated the use of AlignRT alone during DIBH treatment [30] and the use of a goggle to improve patient breathing reproducibility [24]. These studies did not incorporate AlignRT as an automatic or manual beam trigger for gating the DIBH treatment, but rather used it as an imaging tool to confirm patient setup or real-time monitoring. To our knowledge, the presented study is the first to correlate AlignRT data with true target positioning during treatment. It is also the first to evaluate the feasibility of using AlignRT as a surrogate for manual beam control during left breast respiratory-gated DIBH treatment.

We hypothesized that the combined use of RPM to track the respiratory cycle and AlignRT for surface target monitoring could ensure more reproducible patient positioning and a more accurate representation of the chest expansion during DIBH. The primary aim of the study was to evaluate the effectiveness of AlignRT in providing reproducible respiratory gating for left sided breast DIBH treatment. The secondary aims were to investigate the proper threshold level with AlignRT and the translational axis that best correlates with the target motion.

## Materials and Method

### Ethics statement

The study has been approved by the Ohio State University Cancer Institutional Review Board (IRB, 2010C0078). Patient records were made anonymous and de-identified prior to analysis. Patient consent was waived by the IRB for this retrospective study.

### CT simulation and treatment planning

Left-sided breast cancer patients, treated with either lumpectomy or mastectomy, were considered for DIBH treatment. Patients received radiation to the left breast as part of breast conservation therapy or left CW and regional lymph nodes. Target volume and normal structures were contoured following the RTOG breast cancer contouring atlas guidelines. At the time of CT simulation, a free breathing (FB) and a voluntary DIBH scan were acquired for each patient using the GE LightSpeed CT scanner (GE Healthcare, Milwaukee, WI, USA). Following the FB CT, the RPM infrared box was placed on patient's xyphoid process to acquire respiratory motion. The DIBH CT scan was performed using the same isocenter while the patient was instructed to “breathe out, take a deep breath in, and hold it.” The CT scan was acquired when the patient's breathing trace reached the plateau of the respiratory cycle. During the DIBH scan, the RPM trace was recorded, so that it could later be used for beam gating if the DIBH technique was selected for treatment.

Treatment plans were created on both FB and DIBH CTs to provide a dosimetric comparison in target coverage, as well as doses to the heart and lungs, with a prescription of 50 Gy/25 fx for all studied patients. Opposing tangential fields were used for these patients, with field-in-field or electronic compensator techniques to improve dose homogeneity. Ultimate use of DIBH for treatment was determined by the physician based on the dosimetric comparison results.

### Respiratory gating with dual surrogates

The RPM gating system is integrated and operational on the same console as the TrueBEAM system (Varian Medical Systems, Inc., Palo Alto, CA). TrueBEAM also offers integrated on-board kV and MV imaging capabilities, which provide visual anatomical matching to the digitally reconstructed radiograph (DRR) produced using the planning CT image set. Real-time anatomy verification is available through MV Cine acquisition by collecting the exit dose from radiation and forming electronic images. Two levels of beam gating surrogates were adopted, with RPM being the automatic beam control and AlignRT being the secondary gating surrogate for manual beam control during gated treatment.

**Figure 1:**
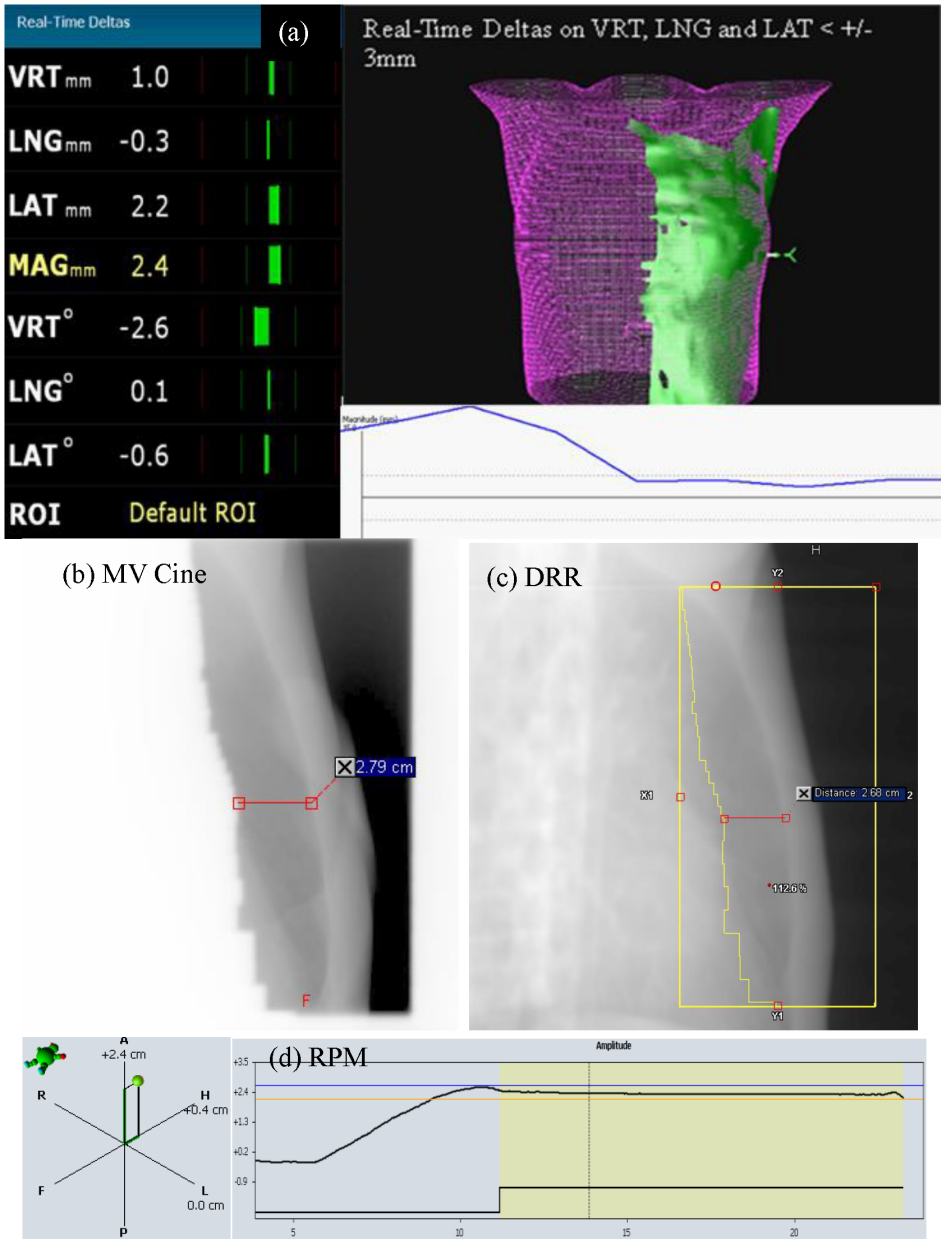
Displays of AlignRT system, MV Cine imaging, DRR, and the RPM system for treatments. (a) AlignRT screen layout showing the RTDs in translational and rotational directions, the overlay of the body rendering from the RSM (pink) and reconstructed surface imaging (green), and the display of the magnitude of all three translational RTDs in mm as a function of time; (b) MV cine image for a left medial field, compared to (c) the DRR of the corresponding field; (d) Patient's RPM trace, as a function of time.

The AlignRT software provides a real-time 3D surface imaging reconstructed from the speckle pattern projected onto the patient's surface, which can then be registered with the planned surface contour (RSM) to provide the patient's real-time positioning offsets (real-time delta, RTD). As shown in Figure 1a, RTD provides a quantitative representation of rigid body transformations in 6 degrees-of-freedom (translational and rotational transformation around three perpendicular axes) registered between the real-time reconstructed surface imaging and the RSM. This occurs at a frame-rate ranging from 0.5 to 1.6 fps (frame per second), depending on the size of surface models and the resolution of ROIs. The magnitude of translational shifts is the vector of lateral, longitudinal and vertical translations, and reported as “MAG”. The display bars of reported RTDs in AlignRT will turn red when the values are beyond the set threshold and green when they are within this threshold. Literature has provided detailed technical information on the AlignRT system [21], [24]–[26].

In our study, the “body” contours from both FB and DIBH CTs were used to create two RSMs, both of which were used for patient positioning. Two ROIs were created for each patient, including the target (breast or chest wall) and the surrounding chest surface, axilla, and the left arm. For patient positioning prior to treatment, the FB RSM was used to align each patient and the DIBH RSM was used to adjust the alignment when the patient was in DIBH. For target monitoring during treatment, the target ROI on DIBH RSM was used to track target motion as a gating surrogate secondary to the RPM. Therapists manually controlled the beam and re-coached patients when the RTDs were beyond the threshold.

Four patients treated with DIBH using the two surrogates were retrospectively analyzed. For the first two patients, one left breast and one left CW, therapists were instructed to solely rely on the RPM and not to make any adjustments according to the AlignRT data. This is a quality assurance process to investigate the correlation between the target and gating surrogates, and to study the proper thresholds for the RTDs. The RTD thresholds serve as a gating window, beyond which the radiation beam should be paused or turned off. The subsequent two patients, also one breast and one CW, were treated with this dual-surrogate protocol with both RPM and AlignRT. Namely, actions were initiated when either RPM or AlignRT signaled out-of-tolerance. Specifically, therapists were instructed to pause the beam and re-coach the patient if RTDs increased beyond the defined threshold based on AlignRT. Furthermore, the same protocol has been adopted in our center for all DIBH treatments, and data was collected for an additional nine breast patients and five CW patients to further prove the efficacy of this protocol.

### Correlation between the target and gating surrogates

Post-treatment data analysis was performed to investigate the correlation between the target position and two gating surrogates, RPM and AlignRT. MV Cine images were taken at the mid-point of beam-on time. Target location at the time of treatment was determined by measuring the CW excursion from one centrally located MLC leaf horizontally to the interior surface of CW on MV cine images (Figure 1b). DRR was evaluated using the lung window template as this was most consistent with the appearance of MV cine images (Figure 1c). The difference between the CW excursions measured on the MV cine image and the DRR was used to determine the accuracy of target positioning. The CW excursion measurement has accuracy to the tenth of a millimeter (mm) but measurement variation could be up to 0.5 mm, due to the MV image quality. The position offset indicated by the RPM surrogate was obtained by subtracting the reference trace plateau value (the reference breath trace at the time of DIBH CT scan) from the RPM amplitude reading at the time of treatment, as shown in Figure 1d. The RPM surrogate position has an accuracy of 1 mm. Therefore, the RPM position offset can be within ±0.5 mm accuracy.

In AlignRT, the RTD coordinates of all translational and rotational directions as well as the translational magnitude (measured in mm), are recorded as a function of time in seconds (Figure 1a). These coordinates coincide with Varian's IEC system, i.e. a positive vertical RTD means the real-time surface imaging (patient's current position) is superior to the RSM (patient's position at time of CT simulation). The magnitude is the vector of all three translational axes, and always recorded as a positive value. The magnitude threshold can be custom defined in the system, which determines the cutoff value for acceptable patent positioning (i.e. at what position the bars change from green to red). The reported RTD values themselves are accurate to the fifth decimal point of one mm. However, the recorded value for RTDs was taken at the mid-point of a timeline during a breath-hold session, and rounded to ±0.5 mm accuracy.

Our hypothesis is that AlignRT better correlates with the actual target position, compared to RPM. To quantify the level of correlation between two subjects, the correlation coefficient is determined by
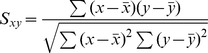



Where x and y are two sets of studied data. *S_xy_* as defined above is a number between -1.0 and +1.0, where -1.0 indicates a strong negative linear correlation and +1.0 indicates a strong positive linear correlation. A larger absolute value of *S_xy_* indicates a higher level of correlation.

## Results

### CT simulations and treatment planning for FB and DIBH

**Figure 2:**
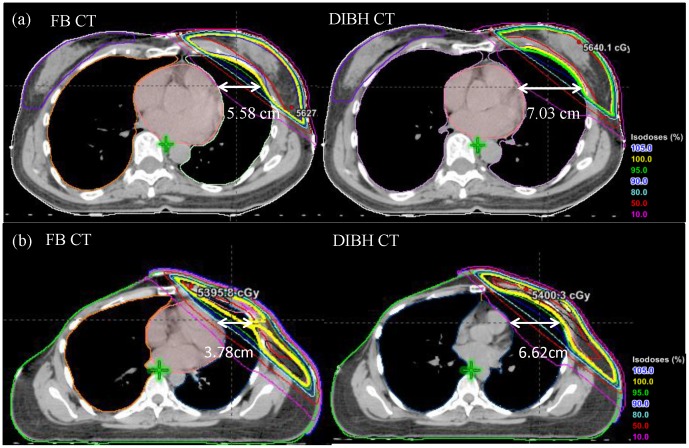
Anatomy and isodose comparisons for (a) the left breast and (b) the left CW patients (left: FB CT; right: DIBH CT). The CW expansion increased from 5.58

Figure 2a and 2b show a left breast and a left CW patient, respectively. The central axial slices (Z = 0 cm) on the FB (left) and DIBH (right) CTs were compared. Measuring at the same coronal level, the CW excursion increases from 5.58 cm on FB to 7.03 cm on DIBH for the left breast patient, resulting in a 60% decrease in maximum heart dose (D_0.1%_) (from 38.2 Gy to 15.1 Gy) and a 85% decrease in mean heart dose (from 5.5 Gy to 0.8 Gy). For the left CW patient, CW excursion at the same coronal level increases from 3.78 cm on FB to 6.62 cm on DIBH, resulting in a 34% decrease in maximum heart dose (D_0.1%_) (from 53.0 Gy to 35.0 Gy) and a 74% decrease in the mean heart dose (from 5.8 Gy to 1.5 Gy). Lung doses from both techniques were comparable.

**Figure 3:**
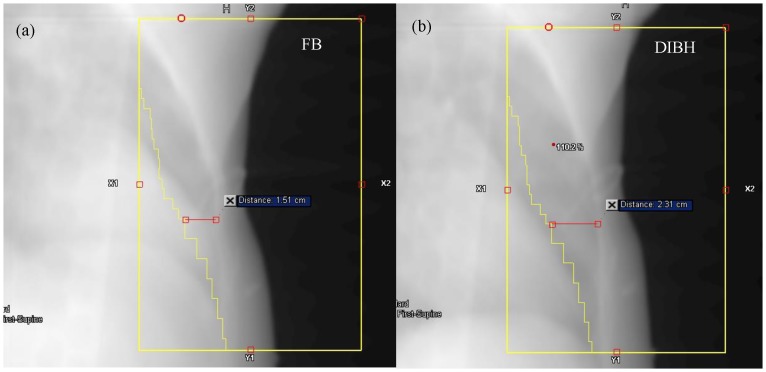
The DRRs of a left medial field on the FB CT (a) and DIBH CT (b). The CW excursion measured from one leaf end to the interior CW surface was increased from 1.51

CW excursion differences can be observed between FB and DIBH when using the beam-eye-view on DRRs (Figure 3). The DRR of a medial beam on the FB CT (Figure 3a) was compared to that on the DIBH CT (Figure 3b). The CW excursions measured from one leaf end to the interior surface of the CW were 1.51 cm on FB and 2.31 cm on DIBH. This measurement can serve as a direct and quantitative representation of target position during treatment.

### Treatment accuracy and correlation between target and RPM surrogate

**Figure 4:**
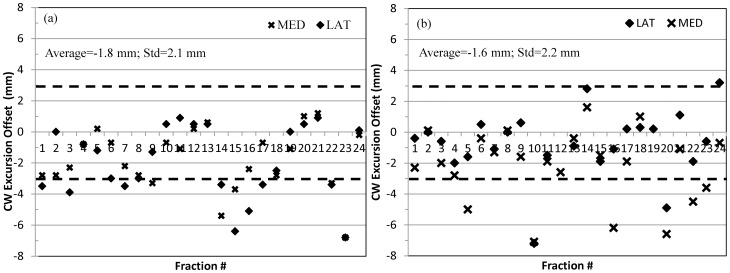
CW excursions offsets of the lateral and medial beams for 24 treatment fractions for the breast patient (a) and CW patient (b), respectively. Dash lines indicate ±3 mm region. The average offset was −1.8 mm with a standard deviation of 2.1 mm for the breast patient. The average offset was −1.6 mm with a standard deviation of 2.2 mm for the CW patient.

CW excursion offsets of the lateral and medial beams for 24 fractions of the first two patients, respectively, were plotted in Figure 4a (breast patient) and 4b (CW patient). MV cine imaging was not performed for the first fraction to avoid imaging complications with verification simulation. For this analysis, offsets within the ±3.0 mm region were considered minimal deviation from the plan. This value was estimated from the data previously presented in literature [15], [32], [33]. As shown in Figure 4a, CW excursion offsets fell within the ±3.0 mm region for most fractions, with an average of −1.8 mm and standard deviation of 2.1 mm. A systematic offset was noted, with the negative average value. The CW excursions measured on the planning DRR for the breast patient were 33.4 mm and 44.8 mm for the lateral and medial beams, respectively. The actual CW excursion varied between 27.3 to 35.0 mm (average = 31.9 mm, standard deviation = 2.4 mm) for the lateral beam and 38.0 to 46.0 mm (average = 43.0 mm, standard deviation = 2.0 mm) for the medial beam. There were several points that resided in (−3.0, −3.5) and (3.0, 3.5) mm regions, which were considered within measurement uncertainties (measurements on the MV cine images have an uncertainty of ±0.5 mm). Seven points (three in medial beams and four in lateral beams) revealed differences larger than ±3.5 mm between CW excursions at the time of treatment and the planning DRR. However, RPM was still within the gate window during these seven beam segments, meaning this potential inaccuracy could not be detected by the RPM system. For the left CW patient, CW excursions measured on the planning DRR were 20.8 mm and 33.5 mm for the lateral and medial beams. The actual CW excursion varied between 14.6 to 25.0 mm (Average = 21.0 mm, standard deviation = 2.2 mm) for the lateral and 27.0 to 35.7 mm (average = 31.9 mm, standard deviation = 2.3 mm) for the medial. As shown in Figure 4b, the average and standard deviation of the overall offsets were −1.6 mm and 2.2 mm, also suggesting the same trend of systematic offset. There were eight points that fell outside the ±3.5 mm region, indicating a possible deviation in the dose delivered to the CW target.

**Figure 5:**
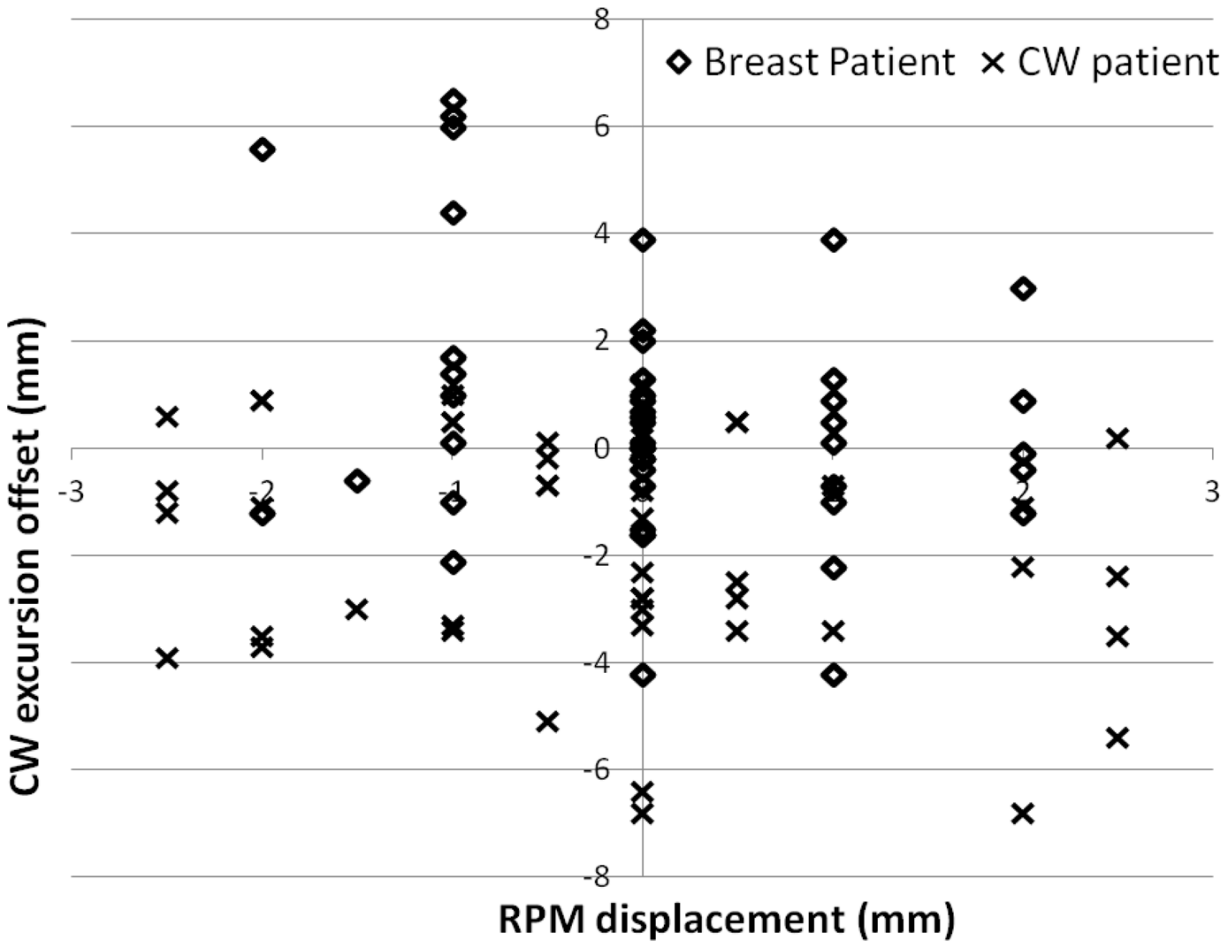
The correlation of RPM displacement (horizontal axis) and CW excursion offset (vertical axis) for the breast and CW patient.

Figure 5 shows the plot of RPM displacements versus CW excursion offsets for both patients. No correlation was found between these two parameters (*S_xy_* is −0.27 for the breast patient and −0.23 for the CW patient). Within the ±2.5 mm RPM gate window, the CW excursion offsets varied from −4.2 mm to 6.5 mm for the breast patient and −6.8 mm to 1.2 mm for the CW patient.

### AlignRT real-time treatment monitoring and its correlation with the target

**Figure 6:**
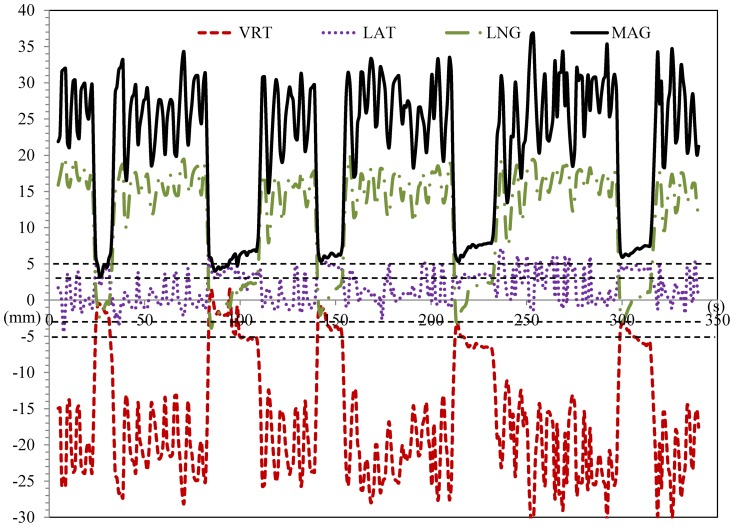
Five segments of breath-holds, corresponding to five beams of one treatment fraction, represented by AlignRT RTDs as a function of time for vertical (red dash line), lateral (purple dotted line), longitudinal (green dash dot line), and magnitude (black solid line). Dash lines indicate ±3 mm and ±5 mm thresholds.

The CW patient was selected for the study of the AlignRT surrogate and its correlation with the CW target due to larger measurement variation of the CW excursion offset as compared to the breast patient. As an example shown in Figure 6, the patient took five deep breaths at the elapsed time intervals of 22 s–32 s, 83 s–110 s, 140 s–154 s, 212 s–233 s, and 298 s–316 s, starting from the first frame. Lateral coordinates showed minimal correlation with the respiratory motion. However, vertical, longitudinal, and magnitude values presented distinct differences between FB and DIBH, correlating to the beam-on times. A superior displacement of 10–22 mm and an anterior displacement of 12–30 mm, varying with the respiratory motion, were recorded from FB to DIBH. This corresponded to the superior and anterior expansions of the CW on the DIBH CT as the patient was taking a deep breath. The superior and anterior displacements between inhalation and exhalation in FB were generally less than 10 mm and varied significantly. Two pairs of horizontal dashed lines show ±3 mm and ±5 mm regions. Of the five segments of breath holds, three were within the ±3 mm region and two were outside. Detailed data analysis was conducted to study individual breath-hold segments of each fraction throughout the treatment.

**Figure 7:**
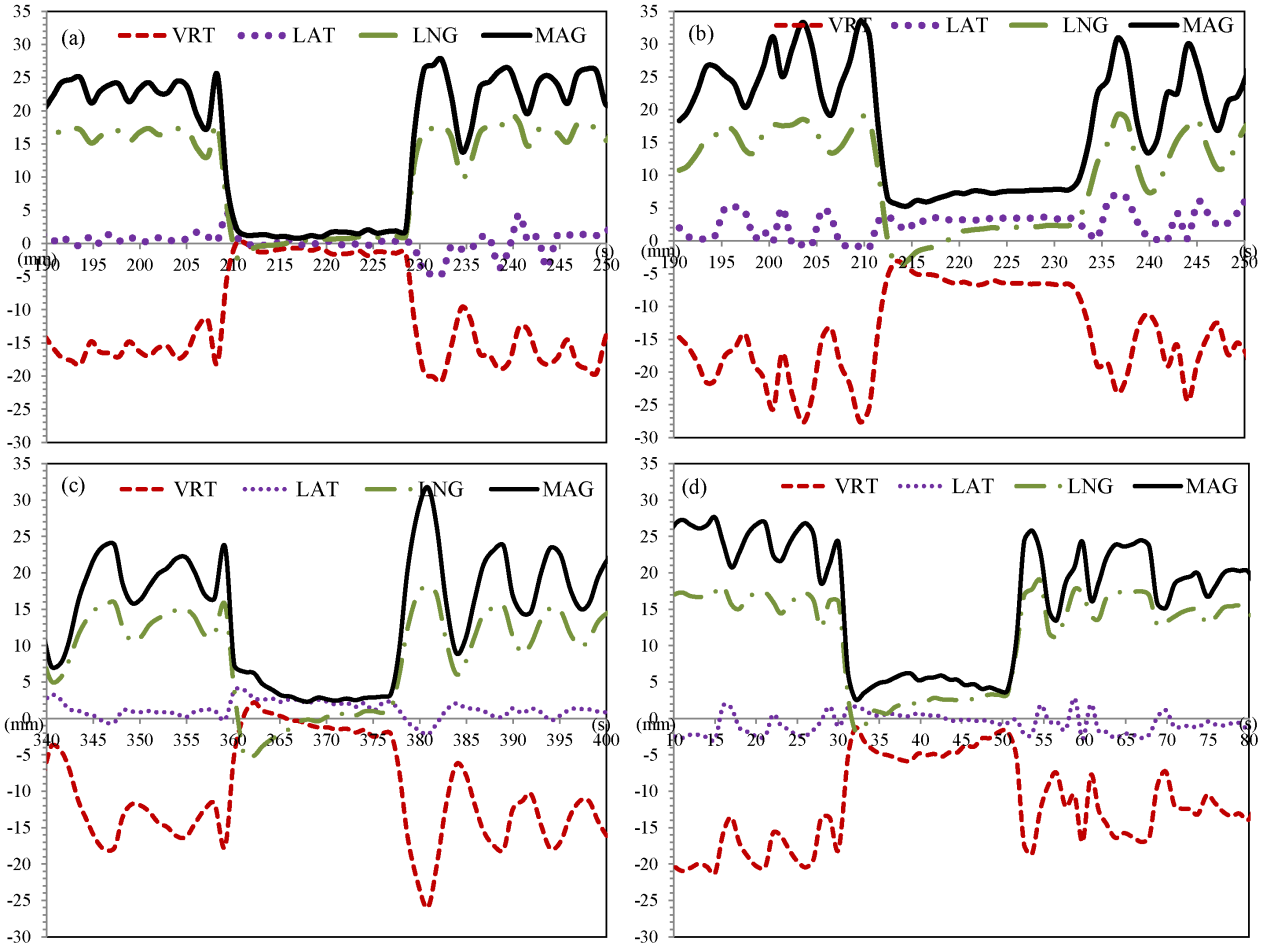
AlignRT RTDs as a function of time for vertical (red dash line), lateral (purple dotted line), longitudinal (green dash dot line), and magnitude (black solid line). Panel (a)–(d) show four different representations of surface motion represented by RTDs.

Figure 7a and 7b display two individual breath-holds between the timeline of 212 s and 233 s. For both treatment fractions, the radiation beam was turned on as the RPM trace entered the gate window. Figure 7a indicates that the RTD values were all within the ±3 mm region during beam-on and post-treatment analysis showed only a 1.6 mm difference in CW excursion between the MV Cine and DRR. However, Figure 7b showed the RTD results of the same beam from a different treatment day. This RTD reported longitudinal offset from −4 to 3 mm and vertical offset from −3 to −7 mm, which resulted in a total offset of 5 to 8 mm in magnitude (Figure 7b). This was mostly due to the decreased CW expansion as the patient may not be able to stably hold the breath and gradually exhaled. The corresponding MV Cine imaging for this beam measured the actual CW excursion at 27.7 mm during treatment, compared to 33.4 mm on the DRR (−5.7 mm discrepancy), which was consistent with the RTD.

**Figure 8:**
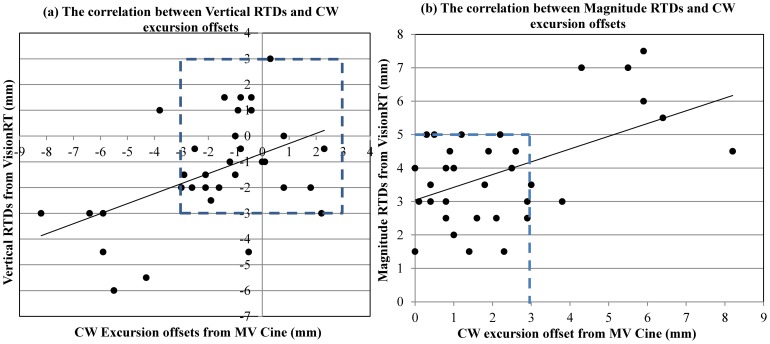
The correlation of the CW excursion offsets with (a) the vertical RTDs and (b) the magnitude RTDs, respectively. A linear correlation was observed with correlation coefficient of 0.47 for the vertical RTDs and 0.52 for the magnitude RTDs. A threshold of ±3 mm for the vertical and 5 mm for the magnitude RTDs can ensure CW excursion offsets to be within ±3 mm.

AlignRT RTD data presented the real-time positional displacement of CW at the time of breath-holding. Figure 7a through 7d show various representations of the intra-fractional motion. Figure 7a shows the ideal case of accurate and stable CW position during breath-hold. However, various changes in CW position during treatment are possible. For example, it can start from negative and drift further down, or start from positive and drift to negative, as demonstrated in Figure 7b and 7c. Furthermore, Figure 7d illustrates another scenario whereby the patient can exhale and inhale during one breath-hold session. For consistency, RTD data for correlation analysis was obtained from the mid-point of each breath-hold session. Figure 8a illustrated a trend of positive linear relationship between the vertical RTDs and CW excursion offsets (*S_xy_* = 0.47). Within the ±3 mm region of vertical RTDs, CW excursion offsets were mostly residing in the ±3 mm region, except for one outlier. The correlation coefficient was slightly higher (*S_xy_* = 0.52) for the magnitude RTD vs. the magnitude of CW excursion offsets, indicating a relatively stronger correlation, as shown in Figure 8b. In the region of the magnitude RTDs < 5 mm, CW excursion offsets resided mostly within ±3 mm. These two figures implied that both vertical and magnitude RTDs can be used as the target surrogate, with a recommended threshold of ± 3 mm and 5 mm, respectively. The magnitude RTD value was considered superior in representing the target motion, due to its higher correlation coefficient with the CW excursion offset.

### DIBH treatment delivery accuracy

**Figure 9:**
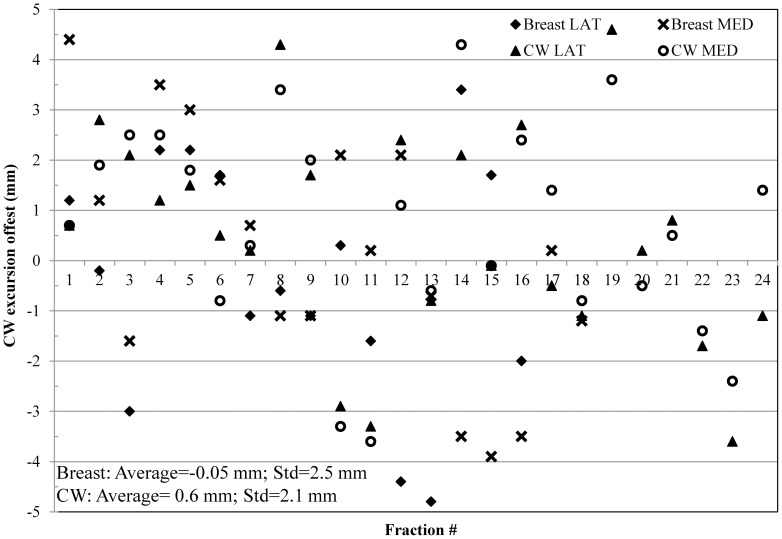
The CW excursion offsets for 24 fractions for the subsequent two patients, one breast and one CW, who was treated with adopting the AlignRT as the secondary surrogate for gating. The dash lines indicate a ±3 mm region. The average offset and its standard deviation were −0.05 mm, 2.5 mm for the breast patient and 0.6 mm, 2.1 mm for the CW patient.

Treatment delivery accuracy was retrospectively analyzed for two patients, one left breast and one left CW, who were treated with the dual-surrogate DIBH protocol. AlignRT was used as the secondary gate surrogate to guide the treatment. Figure 9 displays the CW excursion offsets of both lateral and medial beams for the two patients with a total of 96 data points. Most of points resided within the ±3 mm region. Eight points that were between the regions of 3.0 to 3.5 mm or −3.5 to −3.0 mm were considered within measurement variation. The remaining seven points were within the ±5.0 mm region, possibly due to the uncertainty with the manual gating process. The average offset and its standard deviation were −0.05 mm and 2.5 mm for the breast patient, and 0.6 mm and 2.1 mm for the CW patient. This indicates that the systematic treatment offset can be eliminated with the surface surrogate. Overall treatment time was comparable to regular free-breathing breast treatments.

**Figure 10:**
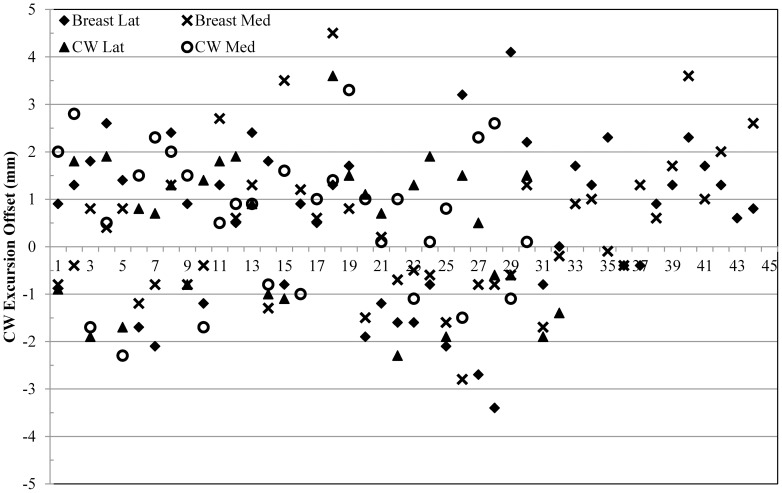
The CW excursion offsets for an additional nine breast patients and five CW patients, who were treated with the new approach combining both RPM and AlignRT as dual surrogates.

To further investigate the improvement with this new approach, we collected CW excursion offset data for an additional nine breast patients and five CW patients. Data was collected on five randomly selected fractions of treatment for each patient, making a total number of 70 data points for both lateral and medial beams, as shown in Figure 10. Most of the offsets were less than ±3 mm. Five were within the uncertainty region (< ±3.5 mm). The other three points were within ±5 mm region. As we gain more experience, the treatment accuracy and target reproducibility have been improved over time, as indicated by Figure 10.

## Discussion

There have been very few studies on the clinical applications of the 3D-surface imaging technique for breast treatment [24], [27]-[30]. Cervino et al. [24] studied the improvements of breathing reproducibility and stability using AlignRT and visual coaching for DIBH. Betgen et al. reported a protocol which involves breath-hold CBCT imaging, 2D fluoroscopy, and AlignRT 3D surface imaging [27]. Alderliesten et al. evaluated the accuracy of the AlignRT system compared with the breath-hold CBCT for the DIBH treatment [28]. Both of the latter studies used breath-hold CBCT as the comparison reference. Tang et al. analyzed and reported treatment accuracy by comparing the chest wall excursions on the port film and the DRR for 50 patients, who had AlignRT as the monitoring tool during treatment [29]. However, there was no AlignRT data reported, nor any correlation between the AlignRT and the true target position. Similarly, Gierga et al. reported the use of AlignRT for real-time breath-hold positioning, without referencing them to the true target positioning [30]. Our present study is the first to investigate the correlation between the target position (as determined by the chest wall excursion) and the AlignRT surface imaging for the left breast DIBH treatment. This study is also the first to demonstrate the lack of correlation between RPM and the target position during DIBH, therefore, supporting the use of AlignRT as a surrogate for the left breast DIBH treatment. Currently, AlignRT is not able to directly interface with a linear accelerator for automated beam control. Therefore, we established an institutional protocol using RPM as the automatic trigger to control the beam at DIBH and AlignRT as the manual trigger to ensure accurate treatment delivery.

Previous reports have demonstrated that there may be a small dosimetric impact on breast or CW coverage with FB respiratory motion, but the impact is negligible for the heart and lung [32], [34], [35]. To reduce breast or CW motion for more accurate radiation targeting, treatment can be performed with inspiratory gating during free breathing; however, this has limited benefits in sparing heart and lung, due to the limited displacement of the breast or CW from the heart during free-breathing. The AlignRT data from our study revealed a 2-fold increase in chest wall expansion during deep inspiration (Figure 6) compared to free-breathing, resulting in movement of heart further from radiation fields. However, the concern with DIBH treatment was the difficulties in target position reproducibility and the consequent inaccuracy in dose delivery. Our investigation identified a lack of correlation between RPM and target positioning for DIBH treatments, indicating a possible limitation of using RPM as the sole gating surrogate. Additional studies are warranted to determine if a new surrogate should be required for DIBH treatment, to ensure accurate dose delivery. The current study proved AlignRT being a more reliable gating surrogate for DIBH due to its improved correlation with breast/CW target position.

AlignRT surface imaging was demonstrated to be a more reliable surrogate than RPM for DIBH treatments. As shown in Figure 5, no correlation was seen between RPM and the breast/CW target position. The RPM alone was not always able to detect intra-fractional positional changes within the set 5 mm gating window. As a result, if RPM is used as the only surrogate for DIBH treatment, there may be deviations in the target positioning, which could result in an unacceptable variation in the accuracy of dose delivery throughout the treatment course. As shown in Figure 4, the average offsets were negative for both patients, suggesting possible deviations in dose delivery to the target. Pepin et al. has reported possible base-line shift with the RPM system [19], which was also observed on several patients during our early experience. This base-line shift is an inherent problem with the RPM and can potentially result in increased dose to the heart. However, the base-line shift can be detected by AlignRT, which indicates the need to reacquire the RPM trace and reestablish the correct baseline.

In addition, the AlignRT system was able to show real-time setup errors (RTDs) that better correlated to patient's anatomy change, as shown in Figure 8. A trend of linear correlation was observed for both vertical and magnitude RTDs, in relationship to the target positioning offset. A higher correlation coefficient (0.47 for vertical RTD and 0.52 for magnitude RTD) with AlignRT suggested that the surface imaging may serve as a better surrogate than RPM. However, these two values only indicated a linear trend, but were not significant to prove any strong linear relationship. Our future area of interest is to have automatic beam-control capability with AlignRT, which should benefit all DIBH treatments and provide accurate patient positioning and monitoring.

The most representative parameter (RTD) for patient monitoring and gating has not previously been reported. Our results suggest that the magnitude RTD seems to be a better monitoring parameter, due to its higher correlation with the target. However, magnitude RTD is always a positive value and is not able to indicate the direction of offset. Therefore, both vertical and magnitude RTDs are recommended for treatment monitoring. The magnitude RTD can identify the need for patient position adjustment and the vertical RTD can determine the direction of adjustment. As determined by the correlation study reported in Figure 8, a gate threshold of ±3 mm and 5 mm is recommended for the vertical and magnitude RTDs, respectively.

Finally, the optimal criteria to determine when DIBH should be chosen over FB for left-sided breast treatment needs to be established. One option is to compare heart doses on both FB and DIBH plans; however, this strategy involves completing two individual plans. Alternatively, the difference in CW expansion between the FB and DIBH CT scans may be a less time-intensive means to determine if DIBH is the superior treatment option. As shown in Figure 2, a minimum of 10 mm difference in CW expansion between FB and DIBH may be used to guide initial clinical judgment regarding the benefit of DIBH at the time of simulation. More patient data is being collected to further evaluate the extent of chest wall excursion and its correlation with reduced heart dose.

## Conclusion

Respiratory-gated DIBH treatment is a viable option to potentially reduce heart dose for left-sided breast cancer patients. To ensure accurate treatment delivery, patient position reproducibility and precise CW expansion during breath hold is essential. Concurrent use with the real-time MV Cine imaging can ensure accurate patient setup and dose delivery, while minimizing the imaging dose to patients. The RPM system alone may not be sufficient for the required level of accuracy in left-sided breast/CW DIBH treatments. AlignRT surface imaging can be used as a new surrogate for patient setup and accurately monitoring inter- and intra- fractional motions. The vertical and magnitude RTDs offer linear correlation with the target position, and can be used as the gating parameter. A threshold of ±3 mm and 5 mm is recommended for the vertical and magnitude RTD, respectively. In conclusion, the target position accuracy of DIBH treatment can be improved by AlignRT in addition to the RPM system.
